# Supplementary material for: High mortality in an outbreak of multidrug resistant Acinetobacter baumannii infection introduced to an oncological hospital by a patient transferred from a general hospital
Source: PLoS One. 2020 Jul 23;15(7):e0234684. doi: 10.1371/journal.pone.0234684 (PMC7377454; doi:10.1371/journal.pone.0234684)
Supplement: S1 File — (DOCX) [file pone.0234684.s001.docx]

**Supplement 1. A method to obtain plasmid profiles from *Acinetobacter***

1. Make an overnight culture in LB medium (3 ml) of your strain starting from one colony
2. Prepare a horizontal gel with 0.75% agarose in TBE 1X buffer (We use gels of 15 cm with and 20 cm length).
3. Determine the DO of the overnight cultures at 600nm. Take enough cells to prepare 1mL of a cell suspension of DO 0.2 and put them into an Eppendorf tube (1.2 mL).
4. Centrifuge sample in a table centrifuge, at 14 000 rpm for 6-8 minutes. Discard the supernatant carefully with a pipette. From this moment, all steps are made on ice**.**
5. Resuspend carefully the pellet in 0.5 mL of sterile cold water using a pipette
6. Add very softly, using the Eppendorf tube wall, 1 mL of a cold solution of 0.3%. sarkosyl (cold). Mix carefully by inversion 5-10 times. Centrifuge sample 6 minutes at 14,000 rpm and discard supernatant. Left pellet on ice.
7. Put the gel made in step 2 in the electrophoresis camera and add enough TBE 1X buffer to the superior edge of the gel. NOT COVER wells with the buffer.
8. Add into each well 30 microliters of a 10% SDS/ 0.1% xylencyanol solution.
9. Run the gel at 100 volts (constant voltage) 15 minutes with inverted polarity.
10. Prepare stock. For each sample add: 40 μL of ficoll + 20 uL lysis solution + 6.7 μL lysozyme at 10 mg/mL.
11. Resuspend pellet carefully and avoiding bubble formation with 60 μL of the stock of step 10.
12. Add buffer to the electrophoresis chamber to cover the gel as you normally do in DNA electrophoresis.
13. In the cold room, resuspend again the pellet carefully and check that samples are homogeneous (an essential step). Load samples in the electrophoresis wells.
14. Run gel with normal polarity at 40 volts for 90 minutes.
15. After that, increase voltage to 100 volts and run the electrophoresis for 15 hours.
16. Stain the gel with 200 mL of an ethidium bromide solution (1 mg/mL) for 15 minutes with gentle agitation (remember that gels of this agarose concentration and this size are VERY fragile).
17. Wash gel in tap water for 15 minutes with gentle agitation. Document gel image.

**Solutions**

TBE 89 mM Tris, 89 mM boric acid, 2 mM EDTA.

Lysozyme 10 mg/Mr. Kept solution in cold room

Ficoll PM400in solution 20% in TRIS EDTA (1mM:10mM, pH8) buffer. Prepare 1 mL aliquots and kept them freeze.

Lysis solution: RNAse A 0.4 mg/mL in water / 0.1% Bromophenol blue. Prepare 300 μL aliquots and kept them freeze.
