# Supplementary material for: High mortality in an outbreak of multidrug resistant Acinetobacter baumannii infection introduced to an oncological hospital by a patient transferred from a general hospital
Source: PLoS One. 2020 Jul 23;15(7):e0234684. doi: 10.1371/journal.pone.0234684 (PMC7377454; doi:10.1371/journal.pone.0234684)
Supplement: S1 Data — (PDF) [file pone.0234684.s002.pdf]

No. Expedien	Edad	Género	Neoplasia	sol=0, hemat	Edo. Actual	rem vs prog
112153	53	1	2	1	1	0
121972	40	1	1	1	3	1
123622	42	0	1	1	1	0
122176	71	0	5	0	1	0
114457	62	1	2	1	1	0
113947	33	1	14	0	1	0
111271	29	0	1	1	1	0
111794	40	1	15	0	4	0
113518	51	0	5	0	1	0
113280	63	0	12	0	1	0
132148	40	1	12	0	3	1
114685	69	1	16	0	1	0
111800	23	1	1	1	1	0
123963	49	0	1	1	1	0
114919	45	1	23	0	21	1
110387	46	1	1	1	1	0
140804	73	0	2	1	1	0
115155	59	0	3	1	1	0
41817	58	0	4	0	4	0
14819	85	1	15	0	4	0
82876	25	1	3	1	2	1
111653	33	0	14	0	1	0
111636	17	1	1	1	1	0
71601	60	1	8	0	2	1
103632	32	0	2	1	3	1
140162	59	0	3	1	1	0
140258	42	1	2	1	3	1
114978	68	0	2	1	1	0
110310	58	1	2	1	5	0
115274	47	0	17	0	1	0
122700	22	1	3	1	2	1
101905	29	1	1	1	3	1
102304	48	1	2	1	2	1
121639	60	1	8	0	1	0
132297	34	1	2	1	1	0
103547	62	1	2	1	4	0
112640	27	1	12	0	1	0
124201	21	0	1	1	3	1
62388	49	0	5	0	3	1
112002	68	0	2	1	1	0
121327	52	1	3	1	1	0

123310	56	0	14	0	1	0
111148	22	1	1	1	3	1
132949	31	1	2	1	1	0
154544	33	1	1	1	1	0
123246	73	0	11	0	1	0
135470	46	1	1	1	1	0
125426	48	0	1	1	1	0
125455	39	0	2	1	1	0
94725	42	1	1	1	3	1
112027	46	0	4	0	1	0
102666	43	1	16	0	2	1
103140	24	1	2	1	2	1
112635	75	0	3	1	2	1
114170	21	0	10	0	1	0
114649	32	1	1	1	1	0
120766	67	1	15	0	1	0
923138	55	0	12	0	1	0
114042	69	1	2	1	1	0
141957	58	1	2	1	1	0
111750	67	1	2	1	1	0
142407	27	1	2	1	1	0
114423	27	0	1	1	2	1
124744	57	0	17	0	1	0
131690	62	1	3	1	5	0
102177	33	1	1	1	5	0
111454	54	0	2	1	1	0
111411	61	1	1	1	3	1
162502	53	0	17	0	1	0
112063	45	1	17	0	1	0
125013	44	0	5	0	5	1
141307	56	1	2	1	1	0
144632	59	1	3	1	5	0
114044	45	1	14	0	1	0
121476	42	1	15	0	1	0
120943	24	1	17	0	1	0
923553	53	0	5	0	1	0
963548	58	1	11	0	4	0
110775	15	0	1	1	1	0
111143	44	0	15	0	1	0
114279	31	0	11	0	1	0
102076	39	0	1	1	1	0
100941	50	0	6	0	3	1

113356	50	1	17	0	1	0
113306	57	1	15	0	1	0
114645	76	0	3	1	1	0
112924	54	0	17	0	1	0
103915	58	1	15	0	4	0
120704	52	1	14	0	1	0
122397	31	0	5	0	2	1
121683	47	0	17	0	3	1
104890	93	1	11	0	1	0
30962	78	0	11	0	4	1
110081	49	1	17	0	2	1
122538	55	0	17	0	1	0
871742	75	0	5	0	4	1
103849	34	1	10	0	2	1
110890	56	1	9	0	1	0
103760	59	1	2	1	4	1
125058	46	0	2	1	1	0
124914	37	1	24	1	2	1
134742	48	0	6	0	1	0
133793	40	1	14	0	1	0
121588	49	0	5	0	5	0
124152	69	0	17	0	1	0
125192	33	1	12	0	2	1

QT recient	Fecha QT	diasqtacinet	RT ?	Comorbilida	(especificar	Hospit previ
0	.	.	0	1	8	1
1	3-Dec-12	146	0	0		1
1	13-Oct-12	32	0	0		1
0	.	.	0	0		1
1	23-Jan-12	10	0	1	38	1
0	.	.	0	1	7	0
1	7-Oct-11	26	0	0		1
0	.		0	1	8	1
0	.		0	0	.	0
0	.		0	0		0
0	.		1	0	.	1
1	1-Dec-11	7	0	1	2	1
0	.	.	0	0	.	0
1	11-Oct-12	15	0	0	.	0
1	21-Jul-12	51	0	0	.	0
0	.	.	0	0	.	1
0	.	.	0	0	.	0
0	.	.	0	0	.	1
0	.	.	0	1	78	0
0	.	.	0	0	.	0
1	12-May-11	62	0	0	.	1
0	.	.	0	0	.	0
1	4-May-11	36	0	0	.	1
0	.	.	0	0	.	0
0	.	.	0	0	.	0
0	.	.	0	0	.	0
1	20-Dec-13	52	1	0	.	1
1	17-Jan-12	13	0	1	12	0
1	16-Feb-11	15	0	1	3	1
0	.	.	0	1	1	1
0	.	.	0	1	5	0
0	.	.	0	1	8	0
1	2-Jan-12	25	0	0	.	1
0	.	.	0	1	3	0
0	.	.	0	0	.	1
0	.	.	1	0	.	0
1	12-Jul-11	14	1	1	8	0
1	27-Feb-13	10	0	1	8	1
1	1-Mar-11	17	0	0	.	0
1	25-Oct-11	23	0	1	12	0
0	.	.	0	1	16	0

0	.	.	0	0	.	0
1	4-Nov-11	15	0	1	1238	1
1	20-Aug-13	12	0	1	3	0
1	1-Nov-15	-1	0	0	.	1
0	.	.	0	1	1	0
1	20-Dec-13	39	0	0	.	1
1	24-Dec-12	30	0	0	0	0
0	.	.	0	0	.	0
1	9-Aug-13	7	0	0	.	1
0	.	.	0	0	.	0
0	.	.	1	1	3	0
1	8-Apr-11	13	1	1	38	1
1	15-Dec-11	41	0	0	.	0
1	14-Feb-12	56	1	0	.	1
1	3-Feb-12	31	0	1	37	1
0	.	.	0	1	12	1
0	.	.	0	0	.	1
1	29-Nov-11	4	0	1	23	0
1	25-May-14	13	0	0		0
0	.	.	0	1	23	0
1	30-Jun-14	9	0	1	58	0
1	2-Jul-12	26	0	0	.	1
0	.	.	0	0	.	0
0	.	.	1	1	1	0
1	24-Jan-11	72	0	0		0
1	22-Apr-11	6	0	1	128	1
1	14-Apr-11	14	0	0	.	1
0	.	.	0	0	.	1
0	.	.	.	1	1	1
0	.	.	0	0	.	0
0	.	.	0	2	.	0
0	.	.	0	8	osteomielitis	0
0	.	.	0	5	.	1
0	.	.	0	0	.	0
0	.	.	0	0	.	1
0	.	.	0	8	ca mama	1
0	.	.	0	0	.	0
0	.	.	0	0	.	0
0	.	.	0	0	.	1
0	.	.	0	0	.	0
1	17-Jul-12	42	0	0	.	1
1	15-Aug-11	16	0	0	.	0

0	.	.	0	3	.	1
0	.	.	0	3	.	1
0	.	.	0	1	.	0
1	.	.	0	2	.	1
0	.	.	0	0	.	1
0	.	.	0	1	.	1
0	.	.	1	0	.	1
1	26-Sep-12	2	0	0	.	0
0	.	.	0	1	.	0
0	.	.	0	0	.	1
1	25-Apr-11	52	0	0	.	1
0	.	.	0	0	.	1
0	.	.	0	1	.	1
0			0	0	.	0
0	.	.	0	0	.	1
1	14-Nov-11	133	0	0	.	1
1	10-Dec-12	13	0	0	.	0
1	29-Dec-14	6	0	0	.	1
1	18-Feb-14	39	0	0	.	1
1	20-Nov-13	15	0	0	.	1
0	.	.	1	1	7	0
0	.	.	0	1	1	1
0	.	.	0	1	7	1

Dias hospit	Que hospital	Dias UTI prev	Infecc previa	Carb previo?	Cefalos prev	absgrmnegp
12	INER	3	0	0	0	1
4	.	.	0	0	0	1
65	.	.	1	1	0	1
11	.	.	0	0	0	0
11	.	.	1	0	1	1
.	.	.	0	0	0	0
21	.	.	1	0	1	1
1	.	.	0	1	1	1
.	.	.	0	0	0	0
.	.	.	0	0	0	0
8	.	.	0	0	0	0
3	.	.	0	0	0	0
.	.	.	0	0	0	0
.	.	.	0	0	0	0
.	.	.	1	0	0	1
5	.	.	0	0	0	0
.	.	.	0	0	0	0
21	incan	1	1	1	0	1
.	.	.	1	0	0	1
.	.	.	0	0	0	0
5	.	.	1	0	1	1
.	.	.	0	0	0	0
7	.	.	1	0	0	1
.	.	.	0	0	0	0
.	.	.	0	0	0	0
.	.	.	0	0	0	0
4	.	.	0	0	0	0
.	.	.	0	0	0	0
7	.	.	0	0	0	0
4	.	.	0	0	0	0
.	.	.	0	0	0	0
.	.	.	0	0	0	0
12	.	.	0	0	0	0
.	.	.	0	0	0	0
10	.	.	0	0	0	0
.	.	.	0	0	0	0
.	.	.	0	0	0	0
27	.	.	0	0	0	0
.	.	.	0	0	0	0
.	.	.	0	0	0	0
.	.	.	1	0	0	0

.	.	.	0	0	0	0
11	.	.	1	0	1	1
.	.	.	0	0	0	0
10	.	.	0	0	0	0
.	.	.	0	0	0	0
27	.	.	1	1	1	1
.	.	.	0	0	0	0
.	.	.	0	0	0	0
20		.	1	0	0	1
.	.	.	0	0	0	0
.	.	.	0	0	0	0
17	.	.	1	0	0	1
.	.	.	1	0	0	1
2	.	.	0	0	0	0
5	.	.	0	0	0	0
5	.	.	1	0	1	1
3	.	.	1	0	0	0
.	.	.	0	0	0	0
.	.	.	0	0	0	0
.	.	.	0	0	0	0
.	.	.	0	0	0	0
7	.	.	0	0	0	0
.	.	.	1	0	0	0
.	.	.	1	0	0	1
.	.	.	0	0	0	0
desc	.	.	0	0	0	0
7	.	.	1	0	0	1
30	.	.	1	0	1	1
desc	.	.	0	0	0	0
.	.	.	0	0	0	0
.	.	.	0	0	0	0
.	.	.	0	0	0	0
desc	.	.	0	0	0	0
.	.	.	0	0	0	0
5	.	.	0	0	0	0
10	.	.	1	0	0	1
.	.	.	0	0	0	0
.	.	.	0	0	0	0
12	incan	8	1	1	1	1
.	.	.	0	0	0	0
20	.	.	0	0	0	0
.	.	.	0	0	0	0

18	.	.	0	0	0	0
9	.	.	0	0	0	0
.	.	.	0	0	0	0
19	.	.	0	1	1	1
17	.	.	1	1	0	1
2	.	.	0	0	0	0
12	.	.	0	0	0	0
.	.	.	0	0	0	0
.	.	.	0	0	0	0
9	.	.	0	0	0	0
47	incan	3	1	1	1	1
7	incan	3	0	0	0	0
7	.	.	0	0	0	0
.	.	.	0	0	0	0
desc	.	.	0	0	0	0
3	.	.	0	0	0	0
.	.	.	0	0	0	0
4	.	.	0	0	0	0
3	.	.	1	0	0	1
22	.	.	1	1	0	1
.	.	.	0	0	0	0
10	.	.	0	0	0	0
desc	.	.	0	0	0	0

Absprevios 1	Dias abs1	Abs previos2	Dias abs pre	Abs previos3	Dias abs pre	Hospitalizad
0	.	0	.	0	.	1
0	.	0	.	0	.	1
12	7	3	7	0	.	1
0	.	0	.	0	.	1
2	10	3	3	16	10	1
0	.	0	.	0	.	1
2	14	3	14	6	10	1
0	.	0	.	0	.	1
0	.	0	.	0	.	1
0	.	0	.	0	.	1
0	.	0	.	0	.	1
0	.	0	.	0	.	1
0	.	0	.	0	.	1
0	.	0	.	0	.	1
4	7	0	.	0	.	1
0	.	0	.	0	.	1
0	.	0	.	0	.	1
12	14	0	.	0	.	1
5	7	0	.	0	.	1
0	.	0	.	0	.	1
1	5	3	5	13	5	1
0	.	0	.	0	.	1
4	13	0	.	0	.	1
0	.	0	.	0	.	1
0	.	0	.	0	.	1
0	.	0	.	0	.	1
0	.	0	.	0	.	1
0	.	0	.	0	.	1
0	.	0	.	0	.	1
0	.	0	.	0	.	1
0	.	0	.	0	.	1
0	.	0	.	0	.	1
0	.	0	.	0	.	1
0	.	0	.	0	.	1
0	.	0	.	0	.	1
0	.	0	.	0	.	1
0	.	0	.	0	.	1
0	.	0	.	0	.	1
0	.	0	.	0	.	1
6	7	0	.	0	.	1
1	nd	0	.	0	.	1

0	.	0	.	0	.	1
2	nd	0	.	0	.	1
0	.	0	.	0	.	1
0	.	0	.	0	.	1
0	.	0	.	0	.	1
2	4	3	3	12	7	1
0	.	0	.	0	.	1
0	.	0	.	0	.	1
7	5	0	.	0	.	1
0	.	0	.	0	.	1
0	.	0	.	0	.	1
2	10	14	14	0	.	1
4	7	5	7	17	7	1
0	.	0	.	0	.	1
0	.	0	.	0	.	1
2	5	13	5	0	.	1
0	.	0	.	0	.	1
0	0	0	0	0	0	1
0	.	0	.	0	.	1
0	.	0	.	0	.	1
0	.	0	.	0	.	1
0	.	0	.	0	.	1
3	8	13	8	0	.	1
5	7	0	.	0	.	1
0	0	0	.	0	.	1
0	0	0	.	0	.	1
2	7	0	.	0	.	1
2	7	5	7	0	.	1
0	0	0	.	0	.	1
0	.	0	.	0	.	1
0	.	0	.	0	.	1
0	.	0	.	0	.	1
0	.	0	.	0	.	0
0	.	0	.	0	.	1
0	.	0	.	0	.	1
3	3	0	.	0	.	1
0	.	0	.	0	.	1
0	.	0	.	0	.	1
12	7	13	10	1	10	1
0	.	0	.	0	.	1
0	.	0	.	0	.	1
0	.	0	.	0	.	1

0	.	0	.	0	.	1
0	.	0	.	0	.	1
0	.	0	.	0	.	1
2	4	3	6	11	11	1
12	16	14	16	8	16	0
0	.	0	.	0	.	1
0	.	0	.	0	.	1
0	.	0	.	0	.	1
0	.	0	.	0	.	1
0	.	0	.	0	.	1
12	19	2	4	3	14	1
0	.	0	.	0	.	1
0	.	0	.	0	.	1
0	.	0	.	0	.	1
0	.	0	.	0	.	1
5	7	0	.	0	.	1
0	.	0	.	0	.	1
0	.	0	.	0	.	1
4	7	0	.	0	.	1
11	5	12	19	3	4	1
0	.	0	.	0	.	1
0	.	0	.	0	.	0
0	.	0	.	0	.	1

Ing Hospit	Egreso hospi	Dias hosp	Neutropenia	Dias neutrop	Motivo ingre	Tx. Empirico
7-Jun-11	15-Jun-11	8	1	5	1	1
28-Mar-13	3-May-13	36	1	50	2	1
8-Nov-12	7-Dec-12	29	0		2	1
25-Jul-12	1-Aug-12	7	0	.	10	1
23-Jan-12	11-Feb-12	19	1	1	3	1
31-Oct-11	21-Dec-11	51	0	.	7	1
7-Oct-11	15-Nov-11	39	0	.	3	1
3-Nov-11	11-Nov-11	8	0	.	7	1
29-Sep-11	7-Oct-11	8	0	.	7	0
7-Sep-11	21-Sep-11	14	0	.	7	1
30-Jan-14	17-Feb-14	18	0	.	15	1
30-Nov-11	11-Dec-11	11	0	.	15	1
12-May-11	29-May-11	17	1	30	1	1
26-Oct-12	4-Nov-12	9	0	.	8	1
1-Jul-12	11-Sep-12	72	0	.	2	1
31-Mar-11	9-Apr-11	9	1	10	14	1
7-Apr-14	14-Apr-14	7	0	.	11	1
28-Mar-12	12-Apr-12	15	0	.	13	1
17-Feb-14	25-Mar-14	36	0	.	4	1
11-Oct-12	22-Nov-12	42	0	.	4	1
15-Jun-11	6-Aug-11	52	1	53	15	1
3-Oct-11	14-Oct-11	11	0	.	7	1
3-May-11	13-Jun-11	41	0	.	1	1
1-Aug-11	30-Aug-11	29	0	.	7	1
25-Apr-11	8-May-11	13	0	.	4	1
23-Jan-14	3-Feb-14	11	0	.	1	1
22-Jan-14	15-Feb-14	24	0	.	1	1
15-Jan-12	16-Apr-12	92	0	.	2	1
21-Jan-11	18-Mar-11	56	0	.	1	1
17-Jan-12	17-Feb-12	31	0	.	11	1
29-Jun-12	10-Aug-12	42	0	.	4	1
4-Jun-12	26-Jun-12	22	0	.	12	1
15-Jan-12	30-Jan-12	15	1	1	3	1
2-Jul-12	14-Jul-12	12	0	.	7	1
31-May-13	6-Jun-13	6	0	.	1	0
27-Jul-11	22-Aug-11	26	0	.	15	0
5-Jul-11	22-Aug-11	48	0	.	1	1
20-Feb-13	10-Mar-13	18	1	8	3	1
4-Mar-11	24-Mar-11	20	0	.	12	1
4-Nov-11	9-Dec-11	35	1	1	10	1
28-Mar-12	8-May-12	41	0	.	1	1

9-Sep-12	13-Oct-12	34	0	.	7	1
27-Oct-11	22-Nov-11	26	1	26	10	1
14-Aug-13	1-Sep-13	18	0	.	14	1
22-Oct-15	5-Nov-15	14	0	.	15	1
27-Aug-12	12-Nov-12	77	0	.	7	1
12-Feb-14	3-Mar-14	19	1	1	3	1
18-Dec-12	3-Apr-13	106	1	60	1	1
20-Dec-12	14-Feb-13	56	0	.	1	1
9-Aug-13	20-Aug-13	11	1	29	3	1
26-May-11	16-Jun-11	21	0	.	1	1
26-Aug-11	1-Sep-11	6	0	.	8	1
23-Mar-11	9-May-11	47	1	4	3	1
6-Jan-12	4-Feb-12	29	0	.	4	1
22-Mar-12	16-May-12	55	0	.	7	1
16-Feb-12	6-Apr-12	50	1	6	3	1
27-Mar-12	1-Jun-12	66	0	.	8	1
11-Jun-14	12-Aug-14	62	0	.	7	1
6-Oct-11	6-Dec-11	61	0	.	1	1
16-May-14	13-Jun-14	28	0	.	14	1
15-May-11	2-Jun-11	18	0	.	8	1
12-Jun-14	11-Jul-14	29	0	.	1	1
15-Jul-12	30-Jul-12	15	0	.	2	1
7-Dec-12	20-Jan-13	44	0	.	4	1
20-Aug-13	20-Sep-13	30	0	.	4	1
12-Feb-11	16-Apr-11	63	0	.	4	1
14-Apr-11	30-Apr-11	16	0	.	1	1
12-Apr-11	30-Apr-11	18	0	.	5	1
10-Jun-16	20-Jun-16	10	0	.	1	1
31-May-11	27-Jun-11	28	0	.	1	1
14-Mar-14	28-Mar-14	14	0	.	15	0
16-Apr-14	12-May-14	26	0	.	2	0
27-Jul-15	11-Aug-15	15	0	.	7	0
.	.	.	0	.	.	0
8-Apr-12	20-Apr-12	12	0	.	1	0
18-May-12	21-May-12	3	0	.	7	0
23-May-12	5-Jun-12	12	0	.	5	1
30-Apr-12	10-May-12	10	0	.	8	0
28-Feb-11	18-May-11	79	1	54	1	1
2-Jun-11	18-Jun-11	16	0	.	15	0
25-Mar-12	8-May-12	44	0	.	7	1
3-Jun-12	5-Jun-12	2	0	-	15	1
17-Aug-11	21-Aug-11	4	0	.	8	0

22-Aug-11	7-Sep-11	16	0	.	1	1
20-Oct-11	13-Nov-11	24	0	.	10	1
27-Jan-12	3-Feb-12	7	0	.	4	1
7-Sep-11	15-Sep-11	8	0	.	10	1
.	.	.	0	.	.	0
15-Aug-12	23-Aug-12	8	0	.	7	1
13-Sep-12	29-Sep-12	16	0	.	12	1
21-Sep-12	27-Sep-12	6	0	.	9	0
5-Jun-11	7-Jul-11	32	0	.	7	1
12-Aug-12	8-Sep-12	27	0	.	10	1
4-Jun-11	12-Jun-11	8	0	.	15	1
20-Aug-12	22-Sep-12	33	0	.	15	0
3-Sep-12	4-Nov-12	31	0	.	15	1
13-Apr-11	5-Jun-11	53	0	.	15	1
7-Mar-11	14-Apr-11	38	0	.	1	1
16-Feb-11	13-Apr-11	56	0	.	4	1
6-Dec-12	23-Dec-12	17	0	.	1	1
26-Dec-12	12-Jan-12	17	0	.	4	1
26-Mar-15	31-Mar-14	5	0	.	10	1
17-Dec-13	30-Dec-13	13	0	.	15	0
12-Feb-13	3-Mar-13	19	0	.	5	1
.	.	.	0	.	.	1
30-Nov-12	11-Dec-12	12	0	.	1	1

Fecha inicio a	absempirgrn	Carbapenem	Dias carbap	Amika	Dias amika	Quin
10/06/2011	1	1	5	1	3	0
28/03/2013	1	1	22	1	3	0
08/11/2012	1	1	3	0	0	0
29/07/2012	1	1	2	1	2	0
23/01/2012	1	1	16	1	2	0
01/11/2011	1	1	20	0	0	0
19/10/2011	1	1	14	1	14	0
03/11/2011	1	1	4	0	0	0
.	0	0	0	0	0	0
08/09/2011	1	1	8	0	0	0
02/02/2014	1	1	12	0	0	0
01/12/2011	1	1	4	0	0	0
21/05/2011	1	1	6	1	7	0
27/10/2012	1	1	5	0	0	0
07/08/2012	1	1	31	1	3	0
31/03/2011	1	1	5	1	9	0
07/04/2014	1	1	6	0	0	0
31/03/2012	1	1	12	0	0	0
17/02/2014	1	1	22	0	0	0
11/10/2012	1	1	14	0	0	0
15/06/2011	1	1	16	1	3	0
03/10/2011	1	1	4	0	0	0
03/05/2011	1	1	25	0	0	0
04/08/2011	1	1	16	1	6	0
25/04/2011	1	1	12	0	0	0
23/01/2014	1	1	12	0	0	0
01/02/2014	1	1	11	0	0	0
22/01/2012	1	1	14	1	12	0
26/01/2011	1	1	28	1	10	0
02/02/2012	1	0	0	0	0	0
29/06/2012	1	0	0	0	0	0
05/06/2012	1	1	6	1	11	0
15/01/2012	1	1	15	0	0	0
09/07/2012	1	1	5	0	0	0
.	0	0	0	0	0	0
02/08/2011	1	1	11	1	3	0
10/07/2011	1	1	14	1	18	0
20/02/2013	1	1	18	1	14	0
06/03/2011	1	1	14	0	0	0
08/11/2011	1	1	17	1	9	0
02/04/2012	1	1	20	0	0	0

11/09/2012	1	1	13	0	0	0
08/11/2011	1	1	12	1	14	0
19/08/2013	1	1	4	1	4	0
22/10/2015	1	1	7	0	0	0
02/09/2012	1	1	27	1	4	1
27/01/2014	1	1	7	1	3	0
25/12/2012	1	1	23	1	10	0
29/12/2012	1	1	43	1	13	0
09/08/2013	1	1	7	1	3	0
26/05/2011	1	1	16	0	0	0
27/08/2011	1	0	0	0	0	0
23/03/2011	1	0	0	1	15	0
07/01/2012	1	1	24	0	0	0
29/03/2012	1	1	25	0	0	0
16/02/2012	1	1	15	1	4	0
27/03/2012	1	1	23	1	18	0
17/06/2014	1	0	0	0	0	0
18/10/2011	1	1	15	0	0	0
04/06/2014	1	1	9	0	0	0
15/05/2011	1	1	15	1	5	0
04/07/2014	1	1	7	0	0	0
15/07/2012	1	1	0	1	8	0
07/12/2012	1	1	16	1	12	0
21/08/2013	1	0	.	0	0	0
12/02/2011	1	1	48	0	0	0
14/04/2011	1	1	12	1	7	0
14/04/2011	1	1	8	0	0	0
11/06/2016	1	0	0	0	0	0
14/06/2011	1	1	7	1	3	0
.	0	0	0	0	0	0
.	0	0	0	0	0	0
	0	0	0	0	0	0
.	0	0	0	0	0	0
.	0	0	0	0	0	0
.	0	0	0	0	0	0
27/05/2012	1	1	17	1	3	0
.	0	0	0	0	0	0
06/03/2011	1	1	36	1	2	0
02/06/2011	1	0	0	0	0	0
26/03/2012	1	1	30	1	7	0
03/06/2012	1	1	12	1	2	0
.	0	0	0	0	0	0

25/08/2011	1	1	9	0	0	0
20/10/2011	1	1	20	0	0	0
30/01/2012	1	0	0	0	0	1
11/09/2011	1	0	0	0	0	0
.	0	0	0	0	0	0
18/08/2012	1	1	5	1	4	0
13/09/2012	1	0	0	0	0	0
.	0	0	0	0	0	0
05/06/2011	1	1	16	0	0	0
27/08/2012	1	1	13	0	0	0
06/06/2011	1	1	6	1	6	0
.	0	0	0	0	0	0
03/09/2015	1	1	16	0	0	0
13/04/2011	1	0	0	1	13	0
16/03/2011	1	0	0	0	0	0
16/02/2011	1	1	14	0	0	0
17/12/2012	1	1	6	1	6	0
01/01/2013	1	1	5	1	5	0
25/03/2014	1	0	0	0	0	0
.	0	0	0	0	0	0
11/02/2013	1	1	15	0	0	0
15/10/2012	1	0	0	0	0	1
30/11/2012	1	1	6	0	0	0

dias quin	C3a	dias c3a	clinda	dias clinda	Metro	Dias metro
0	0	0	0	0	0	0
0	1	11	0	0	0	0
0	1	5	0	0	0	0
0	1	2	0	0	0	0
0	1	4	0	0	0	0
0	1	4	0	0	1	4
0	0	0	1	5	0	0
0	1	3	1	3	0	0
0	0	0	0	0	0	0
0	1	4	0	0	1	4
0	0	0	0	0	0	0
0	1	4	0	0	0	0
0	0	0	0	0	0	0
0	1	3	0	0	1	3
0	1	3	0	0	0	0
0	1	5	0	0	0	0
0	0	0	0	0	0	0
0	0	0	0	0	0	0
0	0	0	0	0	0	0
0	0	0	0	0	0	0
0	1	3	0	0	1	14
0	1	5	0	0	0	0
0	0	0	0	0	0	0
0	1	4	0	0	0	0
0	0	0	0	0	0	0
0	0	0	0	0	0	0
0	0	0	0	0	0	0
0	1	5	0	0	1	9
0	1	9	1	8	0	0
0	1	9	0	0	0	0
0	1	4	0	0	0	0
0	1	11	1	4	0	0
0	0	0	0	0	0	0
0	0	0	0	0	0	0
0	0	0	0	0	0	0
0	1	9	0	0	0	0
0	1	10	0	0	0	0
0	0	0	0	0	1	5
0	0	0	1	10	0	0
0	0	0	0	0	1	3
0	0	0	0	0	0	0

0	1	3	0	0	1	14
0	0	0	0	0	1	14
0	0	0	0	0	0	0
0	0	0	0	0	0	0
15	1	15	1	5	0	0
0	1	3	0	0	0	0
0	1	4	1	5	0	0
0	0	0	0	0	0	0
0	1	3	0	0	1	10
0	1	5	1	3	0	0
0	1	3	0	0	0	0
0	1	18	0	0	0	0
0	0	0	0	0	0	0
0	0	0	0	0	0	0
0	1	4	0	0	1	13
0	1	9	1	10	0	0
0	1	6	0	0	1	6
0	1	9	0	0	0	0
0	0	0	0	0	0	0
0	0	0	0	0	0	0
0	0	0	0	0	1	7
0	1	5	0	0	1	8
0	1	5	0	0	1	7
0	1	10	0	0	0	0
0	1	5	0	0	0	0
0	1	8	0	0	1	5
0	1	6	0	0	0	0
0	0	0	0	0	0	0
0	1	6	1	3	0	0
0	0	0	0	0	0	0
0	0	0	0	0	0	0
0	0	0	0	0	0	0
0	0	0	0	0	0	0
0	0	0	0	0	0	0
0	0	0	0	0	0	0
0	0	0	0	0	0	0
0	0	0	0	0	0	0
0	1	2	0	0	0	0
0	1	4	1	4	0	0
0	0	0	0	0	0	0
0	0	0	0	0	0	0
0	0	0	0	0	0	0

0	1	4	0	0	0	0
0	0	0	0	0	0	0
7	1	4	0	0	0	0
0	1	4	1	3	0	0
0	0	0	0	0	0	0
0	0	0	0	0	0	0
0	1	3	1	3	0	0
0	0	0	0	0	0	0
0	1	10	0	0	1	10
0	0	0	0	0	0	0
0	0	0	0	0	0	0
0	0	0	0	0	0	0
0	1	2	0	0	1	2
0	1	13	0	0	0	0
0	1	14	0	0	0	0
0	1	5	0	0	0	0
0	0	0	0	0	0	0
0	1	5	0	0	0	0
0	1	3	0	0	0	0
0	0	0	0	0	0	0
0	0	0	0	0	0	0
16	0	0	0	0	0	0
0	1	6	1	6	0	0

Vanco	Dias vanco	Claritro	Dias clarit	TMP	Dias TMP	Line
0	0	0	0	0	0	0
1	1	0	0	0	0	0
0	0	1	5	1	3	0
0	0	0	0	0	0	0
0	0	0	0	0	0	0
1	17	0	0	0	0	1
1	7	0	0	0	0	0
0	0	0	0	0	0	0
0	0	0	0	0	0	0
0	0	0	0	0	0	0
0	0	0	0	0	0	0
0	0	0	0	0	0	0
0	0	0	0	0	0	0
0	0	0	0	0	0	0
1	9	0	0	0	0	0
1	5	1	2	0	0	0
1	6	0	0	0	0	1
0	0	0	0	0	0	0
1	11	1	15	0	0	0
0	0	0	0	0	0	0
1	14	0	0	0	0	0
0	0	0	0	0	0	0
1	14	0	0	1	27	0
1	11	0	0	0	0	0
0	0	1	17	0	0	0
0	0	0	0	0	0	0
1	10	1	7	1	3	0
1	18	0	0	0	0	0
1	14	0	0	0	0	0
0	0	0	0	0	0	0
0	0	1	6	1	28	0
1	6	0	0	0	0	0
1	13	0	0	0	0	0
0	0	0	0	0	0	0
0	0	0	0	0	0	0
1	6	0	0	1	6	0
1	3	0	0	0	0	0
0	0	0	0	0	0	0
1	6	0	0	0	0	0
0	0	0	0	1	13	0
1	25	0	0	0	0	0

1	6	0	0	0	0	0
0	0	0	0	0	0	0
0	0	0	0	0	0	0
1	4	0	0	0	0	0
1	18	0	0	0	0	0
0	0	0	0	0	0	0
1	20	0	0	0	0	0
1	12	0	0	0	0	0
0	0	0	0	0	0	0
1	10	0	0	0	0	0
0	0	0	0	0	0	0
1	14	0	0	0	0	0
1	7	0	0	1	10	0
1	18	0	0	0	0	0
1	3	0	0	0	0	0
1	9	0	0	0	0	1
0	0	0	0	0	0	0
1	7	0	0	0	0	0
1	9	0	0	0	0	0
1	5	0	0	0	0	0
0	0	1	7	1	7	0
0	0	0	0	1	7	0
0	0	1	5	1	15	0
0	0	1	15	1	15	0
1	48	1	9	1	44	0
1	3	0	0	0	0	0
1	10	0	0	0	0	0
0	0	1	7	1	7	0
1	7	0	0	0	0	0
0	0	0	0	0	0	0
0	0	0	0	0	0	0
0	0	0	0	0	0	0
0	0	0	0	0	0	0
0	0	0	0	0	0	0
0	0	0	0	0	0	0
0	0	0	0	0	0	0
0	0	0	0	0	0	0
1	36	0	0	0	0	0
0	0	0	0	0	0	0
1	30	0	0	0	0	0
1	5	0	0	13	0	0
0	0	0	0	0	0	0

1	4	0	0	0	0	0
1	5	0	0	1	14	0
0	0	1	4	0	0	0
0	0	0	0	0	0	0
0	0	0	0	0	0	0
0	0	0	0	0	0	0
1	4	0	0	0	0	0
0	0	0	0	0	0	0
0	0	0	0	0	0	0
0	0	0	0	0	0	0
1	6	0	0	0	0	0
0	0	0	0	0	0	0
0	0	0	0	0	0	0
1	7	0	0	0	0	0
1	14	0	0	0	0	0
0	0	1	10	0	0	0
1	1	0	0	0	0	0
0	0	1	5	0	0	0
0	0	0	0	0	0	0
0	0	0	0	0	0	0
0	0	0	0	0	0	1
0	0	0	0	0	0	0
0	0	0	0	0	0	0

Diaz line	Pip/taz	dias pip/taz	Dicloxa	dias dicloxa	Ingreso a UC	Fecha ing UC
0	0	0	0	0	0	.
0	0	0	0	0	1	28-Apr-13
0	0	0	0	0	1	11-Nov-12
0	0	0	0	0	1	29-Jul-12
0	0	0	0	0	1	29-Jan-12
20	0	0	0	0	1	1-Nov-11
0	0	0	0	0	1	19-Oct-11
0	0	0	0	0	1	4-Nov-11
0	0	0	0	0	1	30-Sep-11
0	0	0	0	0	1	11-Sep-11
0	1	3	0	0	1	7-Feb-14
0	0	0	0	0	1	2-Dec-11
0	0	0	0	0	1	23-May-11
0	0	0	0	0	1	27-Oct-12
0	0	0	0	0	1	2-Sep-12
0	0	0	0	0	1	4-Apr-11
0	0	0	0	0	1	8-Apr-14
0	0	0	0	0	1	31-Mar-12
0	0	0	0	0	1	19-Feb-14
0	0	0	0	0	1	13-Oct-11
0	0	0	0	0	0	.
0	0	0	0	0	1	4-Oct-11
0	0	0	0	0	1	20-May-11
0	0	0	0	0	1	4-Aug-11
0	0	0	0	0	1	25-Apr-11
0	0	0	0	0	1	3-Feb-14
1	3	0	0	0	1	4-Feb-14
0	0	0	0	0	1	1-Feb-12
0	0	0	0	0	1	26-Jan-11
0	0	0	0	0	1	20-Jan-12
0	0	0	0	0	1	3-Jul-12
0	0	0	0	0	0	.
0	0	0	0	0	1	21-Jan-12
0	0	0	0	0	1	13-Jul-12
0	0	0	0	0	0	.
0	0	0	0	0	1	16-Aug-11
0	0	0	0	0	1	10-Jul-11
0	0	0	0	0	0	.
0	0	0	0	0	1	6-Mar-11
0	0	0	0	0	1	9-Nov-11
0	0	0	0	0	1	11-Apr-12

0	0	0	0	0	1	11-Sep-12
0	0	0	0	0	1	21-Nov-11
1	9	0	0	0	1	1-Sep-13
0	0	0	0	0	1	23-Oct-15
0	0	0	0	0	1	2-Sep-12
0	0	0	0	0	0	.
0	0	0	0	0	1	13-Jan-13
0	0	0	0	0	1	27-Dec-12
0	1	9	0	0	0	.
0	0	0	0	0	1	27-May-11
0	0	0	0	0	1	27-Aug-11
0	1	3	0	0	0	.
0	0	0	1	8	1	7-Jan-12
0	0	0	0	0	1	29-Mar-12
0	0	0	0	0	1	17-Feb-12
8	0	0	0	0	1	2-Apr-12
0	0	0	0	0	1	25-Jun-14
0	0	0	0	0	1	13-Nov-11
0	0	0	0	0	1	3-Jun-14
0	0	0	0	0	1	15-May-11
0	1	3	0	0	0	.
0	0	0	0	0	0	.
0	0	0	0	0	1	11-Dec-12
0	1	4	0	0	1	23-ago-13 y (
0	0	0	0	0	1	23-Feb-11
0	0	0	0	0	1	21-Apr-11
0	0	0	0	0	0	.
0	1	2	0	0	0	.
0	0	0	0	0	0	.
0	0	0	0	0	0	.
0	0	0	0	0	0	.
0	0	0	0	0	0	.
0	0	0	0	0	0	.
0	0	0	0	0	0	.
0	0	0	0	0	0	.
0	0	0	0	0	0	.
0	0	0	0	0	0	.
0	0	0	0	0	0	.
0	0	0	0	0	0	.
0	0	0	0	0	1	26-Mar-12
0	0	0	0	0	1	22-Aug-12
0	0	0	0	0	0	.

0	0	0	0	0	0	.
0	0	0	0	0	1	21-Oct-11
0	0	0	0	0	0	.
0	0	0	0	0	0	.
0	0	0	0	0	0	.
0	0	0	0	0	0	.
0	0	0	0	0	0	.
0	0	0	0	0	0	.
0	0	0	0	0	1	6-Jun-11
0	0	0	0	0	1	14-Aug-12
0	0	0	0	0	0	.
0	0	0	0	0	0	.
0	0	0	0	0	0	.
0	0	0	1	15	0	.
0	0	0	0	0	0	.
0	0	0	0	0	1	19-Feb-11
0	0	0	1	4	1	23-Dec-12
0	0	0	0	0	0	.
0	0	0	0	0	0	.
0	0	0	0	0	1	27-Dec-13
10	0	0	0	0	1	13-Feb-13
0	0	0	0	0	0	.
0	0	0	0	0	0	.

Fecha egreso	diasuci	SOFA ing	sofa 1=10ma	APACHE ing	apache 1=20	diasingacine
.	.	.	.	.	.	3
3-May-13	5	13	1	15	0	31
30-Nov-12	19	7	0	20	1	6
1-Aug-12	3	4	0	22	1	5
11-Feb-12	13	8	0	15	0	10
6-Dec-11	35	3	0	15	0	7
31-Oct-11	12	12	1	17	0	26
8-Nov-11	4	3	0	8	0	3
4-Oct-11	4	4	0	13	0	4
15-Sep-11	4	2	0	15	0	7
11-Feb-17	4	5	0	6	0	18
11-Dec-11	9	8	0	22	1	8
29-May-11	6	5	0	13	0	14
1-Nov-12	5	6	0	20	1	1
11-Sep-12	9	11	1	17	0	71
9-Apr-11	5	11	1	30	1	0
13-Apr-14	5	9	0	22	1	3
12-Apr-12	12	12	1	27	1	14
21-Mar-14	30	9	0	11	0	16
15-Nov-11	33	4	0	14	0	6
.	.	.	.	.	.	28
12-Oct-11	8	10	1	15	0	7
18-Jun-11	29	4	0	23	1	37
26-Aug-11	22	9	0	13	0	23
8-May-11	13	6	0	5	0	13
3-Feb-14	1	15	1	31	1	11
15-Feb-14	11	8	0	11	0	19
1-Mar-12	29	8	0	21	1	15
2-Mar-11	35	7	0	16	0	41
21-Jan-12	2	1	0	11	0	17
10-Aug-12	38	11	1	18	0	30
.	.	.	.	.	.	21
30-Jan-12	9	12	1	16	0	12
14-Jul-12	1	5	0	28	1	11
.	.	.	.	.	.	3
22-Aug-11	6	9	0	16	0	22
30-Jul-11	20	12	1	14	.	21
.	.	.	.	.	.	17
12-Mar-11	6	4	0	10	0	14
29-Nov-11	20	12	1	14	0	13
1-May-12	20	11	1	22	1	23

3-Oct-12	22	6	0	12	0	11
21-Nov-11	0	12	1	25	1	23
1-Sep-13	0	11	1	18	0	18
5-Nov-15	13	10	1	18	0	9
2-Oct-12	30	9	0	22	1	35
.	.	.	.	.	.	1
10-Feb-13	28	4	0	19	0	36
24-Jan-13	27	5	0	10	0	18
.	.	.	.	.	.	7
15-Jun-11	19	7	0	16	0	12
31-Aug-11	4	7	0	10	0	4
.	.	.	.	.	.	30
3-Feb-12	27	8	0	28	1	19
28-Apr-12	30	9	0	13	0	19
16-Mar-12	28	12	1	27	1	17
20-May-12	48	9	0	21	1	15
9-Jul-14	14	8	0	20	1	38
20-Nov-11	7	6	0	27	1	58
13-Jun-14	10	13	1	16	0	22
2-Jun-11	18	7	0	25	1	10
.	.	.	.	.	.	27
.	.	.	.	.	.	13
6-Jan-13	26	11	1	15	0	34
29-ago-13 y2	19	5	0	12	0	26
3-apr-11	39	9	0	15	0	56
30-Apr-11	9	9	0	.	.	14
.	.	.	.	.	.	16
.	.	.	.	.	.	1
.	.	.	.	.	.	.
.	.	.	.	.	.	.
.	.	.	.	.	.	.
.	.	.	.	.	.	.
.	.	.	.	.	.	.
.	.	.	.	.	.	.
.	.	.	.	.	.	.
.	.	.	.	.	.	.
.	.	.	.	.	.	.
.	.	.	.	.	.	.
.	.	.	.	.	.	.
4-Apr-12	9	3	0			11
8-Oct-12	47	10	1	.	.	38
.	.	.	.	.	.	.

.	.	.	.	.	.	.
6-Nov-11	16	8	0			
.	.	.	.			
.	.	.	.	.	.	.
.	.	.	.	.	.	.
.	.					
.	.	.	.	.	.	
.	.	.	.	.	.	.
24-Jun-11	14	5	0			
20-Aug-12	6	7	0			
.	.	.	.	.		
.	.	.	.	.	.	.
.	.	.	.			
.	.					
.	.	.	.			
3-Apr-11	43	7	0			
23-Dec-12	0	15	1			17
.	.	.	.			8
.	.	.	.			3
30-Dec-13	3	4	0			-12
23-Feb-13	10	15	1			19
.	.	.	.			.
.	.	.	.			4

diasuciacine	Fecha	Dias abs acir	Fecha acinet	Sitio aislami	dias acineto	1=hemo 0=b
.	11	4	14-Jun-11	1	0	1
0	13	31	28-Apr-13	2	0	0
3	12	6	14-Nov-12	2	3	0
1	12	1	30-Jul-12	3	1	1
4	12	10	2-Feb-12	2	4	0
13	11	6	7-Nov-11	2	6	0
13	11	14	2-Nov-11	2	14	0
3	11	3	6-Nov-11	2	2	0
3	11	.	3-Oct-11	2	3	0
3	11	6	14-Sep-11	2	3	0
6	14	15	17-Feb-14	2	10	0
2	11	7	8-Dec-11	1	6	1
3	11	5	26-May-11	1	3	1
0	12	0	27-Oct-12	2	0	0
8	12	34	10-Sep-12	2	8	0
6	11	4	4-Apr-11	2	0	0
2	14	3	10-Apr-14	2	2	0
11	12	11	11-Apr-12	1	11	1
14	14	16	5-Mar-14	2	14	0
4	12	6	17-Oct-12	2	370	0
.	11	28	13-Jul-11	2		0
6	11	7	10-Oct-11	2	6	0
20	11	37	9-Jun-11	2	20	0
20	11	20	24-Aug-11	2	20	0
13	11	13	8-May-11	1	13	1
-2	14	9	1-Feb-14	3	-2	1
6	14	9	10-Feb-14	1	6	1
-2	12	8	30-Jan-12	3	-2	1
34	11	36	3-Mar-11	1	36	1
14	12	1	3-Feb-12	1	14	1
26	12	30	29-Jul-12	1	26	1
.	12	18	23-Jun-12	1		1
6	12	12	27-Jan-12	3	6	1
0	12	4	13-Jul-12	3	0	1
.	13	.	3-Jun-13	1		1
2	11	16	18-Aug-11	2	2	0
16	11	16	26-Jul-11	2	16	0
.	13	17	9-Mar-13	1		1
12	11	12	18-Mar-11	2	12	0
8	11	9	17-Nov-11	3	8	1
9	12	18	20-Apr-12	2	9	0

9	12	9	20-Sep-12	2	9	0
2	11	11	19-Nov-11	1	-2	1
0	13	13	1-Sep-13	2	0	0
8	15	9	31-Oct-15	2	8	0
10	12	29	1-Oct-12	2	29	0
.	14	1	28-Jan-14	2		0
10	13	29	23-Jan-13	2	10	0
11	13	9	7-Jan-13	2	11	0
.	13	7	16-Aug-13	1		1
11	11	12	7-Jun-11	2	11	0
3	11	3	30-Aug-11	2	3	0
.	11	29	21-Apr-11	1		1
18	12	18	25-Jan-12	2	18	0
12	12	12	10-Apr-12	2	12	0
16	12	18	5-Mar-12	2	17	0
9	12	15	11-Apr-12	2	9	0
24	14	32	19-Jul-14	2	24	0
19	11	46	17-Nov-11	2	4	0
4	14	3	7-Jun-14	2	4	0
10	11	10	25-May-11	1	10	1
.	14	5	9-Jul-14	2		0
.	12	13	28-Jul-12	1		1
30	13	35	11-Jan-13	2	31	0
22	13	25	15-Sep-13	2		0
43	11	56	7-Apr-11	2	43	0
7	11	14	26-Apr-11	2	7	0
.	11	14	27-Apr-11	1		0
.	15	1	10-Jun-15	2	.	0
.	11	2	12-Jun-11	2	.	0
.	14	.	15-Mar-14	3	.	2
.	14	.	16-Apr-14	5	.	0
.	15	.	27-Jul-15	7	.	2
.	14	.	11-Oct-11	5	.	0
.	12	.	30-Apr-12	5	.	0
.	12	.	18-May-12	7	.	2
.	12	0	25-May-12	5	.	0
.	12	.	9-May-12	5	.	0
.	11	50	26-Apr-11	5	.	0
.	11	.	5-Jun-11	5	.	0
11	12	11	5-Apr-12	5	.	0
7	12	16	29-Aug-12	5	.	0
.	11	.	31-Aug-11	5	.	0

.	11	-1	24-Aug-11	7	.	2
	11	17	6-Nov-11	2	17	0
	12	3	2-Feb-12	2	.	2
.	11	0	11-Sep-11	5	.	2
.	11	.	22-Jul-11	3	.	2
	12	5	23-Aug-12	5	.	2
	12	1	14-Sep-12	3	.	2
.	<	.	25-Sep-12	5	.	2
	11	23	28-Jun-11	5	-22	2
	12	-3	11-Aug-12	5	3	2
	11	0	6-Jun-11	5	.	2
.	12	.	13-Aug-12	5	.	2
	12	23	26-Sep-12	5	.	2
	11	30	13-May-11	3	.	2
	11	17	2-Apr-11	3	.	2
	11	0	27-Mar-11	5	36	2
0	12	0	23-Dec-12	2	0	0
0	13	1	4-Jan-13	7	.	2
	14	4	29-Mar-14	7	.	2
-22	13	.	5-Dec-13	7	.	2
18	13	19	3-Mar-13	3	.	2
.	12	0	15-Oct-12	5	.	2
.	12	4	3-Dec-12	7	.	2

no. aislamie	Sitio dos aisl	Colon/Infec	colbronq=0,	solo bronq y	SOFA cultivo	sofacult10m
		1	2	2	8	0
		0	0	.	13	1
		1	1	1	6	0
		1	1	1	11	1
2	10	1	1	1	9	0
3	350	1	1	1	11	1
2	10	0	0	.	2	0
		0	0	.	3	0
		0	0	.	3	0
		0	0	.	2	0
		1	1	1	11	1
2	10	1	2	2	8	0
		1	2	2	5	0
		1	1	1	6	0
		1	1	1	13	1
		1	1	1	11	1
		1	1	1	9	0
		1	2	2	14	1
		1	1	1	3	0
		1	1	1	5	0
		0	0	.	1	0
		0	0	.	0	0
		1	1	1	5	0
		0	0	.	3	0
		1	2	2	4	0
2	10	1	1	1	15	1
		1	2	2	11	1
2	10	1	1	1	12	1
2	10	1	2	2	0	0
		1	2	2	1	0
		1	2	2	8	0
		1	2	2	4	0
2	10	1	1	1	11	1
		1	1	1	5	0
		1	2	2	0	0
2	10	1	1	1	9	0
2	30	1	1	1	7	0
		1	2	2	3	0
		0	0	.	0	0
		1	1	1	8	0
		1	1	1	10	1

		1	1	1	0	0
		1	2	2	12	1
		1	1	1	11	1
		1	1	1	6	0
		0	0	.	2	0
		0	0	.	5	0
		0	0	.	0	0
3	350	0	0	.	2	0
		1	2	2	8	0
		1	1	1	9	0
		0	0	.	4	0
		1	2	2	3	0
		1	1	1	13	1
2	50	1	1	1	7	0
2	10	1	1	1	12	1
		1	1	1	9	0
2	50	1	1	1	9	0
		0	0	.	1	0
		1	1	1	13	1
		1	2	2	2	0
		1	1	1	9	0
		1	2	2	3	0
		0	0	.	0	0
		1	1	1	6	0
3	130	0	0	.	0	0
		1	1	1	14	1
		1	2	2	12	1
		1	1	1	2	0
		1	1	1	0	0
		1	3	.	2	0
		1	1	1	0	0
		1	3	.	3	0
		0	0	.	2	0
		0	0	.	0	0
		0	3	.	0	0
2	30	0	0	.	0	0
		0	0	.	0	0
		0	0	.	1	0
		1	1	1	1	0
		1	1	1	3	0
		1	1	1	6	0
		0	0	.	0	0

		0	3	3	0	0
		0	0	.	3	0
		0	0	.	3	0
		0	3	.	3	0
		0	2	2	0	0
		0	3	.	0	0
		1	3	.	3	0
		0	3	.	0	0
		0	3	.	1	0
		0	3	.	0	0
2	53	0	2	2	0	0
		1	2	2	1	0
		0	2	2	0	0
		1	3	.	0	0
		0	3	.	0	0
		0	3	.	2	0
		1	1	1	15	1
		0	3	.	0	0
		1	3	.	9	0
		1	3	.	0	0
		0	3	.	0	0
		0	3	.	0	0
		1	3	.	0	0

APACHE cult	apachecult2(	Abs Acinetol	abs apropiac	colistmas48l	fecha inicio (	dias colis
20	1	0	0	0		
15	0	0	0	0		
13	0	1	1	1	16-Nov-12	15
27	1	0	0	0		
16	0	1	1	1	3-Feb-12	7
17	0	1	1	1	11-Nov-11	7
8	0	0	0	0		
6	0	0	0	0		
13	0	0	0	0		
9	0	0	0	0		
15	0	0	0	0		
15	0	0	0	0		
23	1	0	0	0		
20	1	0	0	0		
18	0	0	0	0		
30	1	0	0	0		
20	1	0	0	0		
19	0	0	0	0		
12	0	1	1	1	6-Mar-14	14
14	0	1	1	1	19-Oct-12	18
.	.	0	0	0		
20	1	0	0	0		
17	0	0	0	0		
13	0	0	0	0		
20	1	0	0	0		
31	1	0	0	0		
20	1	1	1	1		4
25	1	1	1	1	1-Feb-12	17
.	0	0	0	0		
14	0	1	1	0		
14	0	1	1	1		24
.	.	0	0	0		
14	0	1	1	1	27-Jan-12	3
28	1	0	0	0		
nd	.	0	0	0		
9	0	0	0	0		
14	0	0	0	0		
.	.	0	0	0		
.	.	0	0	0		
23	1	0	0	0		
5	0	1	1	1	20-Apr-12	18

9	0	1	1	1	27-Sep-12	16
27	1	0	0	0		
18	0	0	0	0		
18	0	1	1	1		3
14	0	0	0	0		
16	0	0	0	0		
12	0	0	0	0		
12	0	0	0	0		
29	1	0	0	0		
25	1	0	0	0		
6	0	0	0	0		
.	.	1	1	0		
28	1	1	1	1		5
12	0	1	1	1	15-Apr-12	29
27	1	1	1	0		
22	1	1	1	1	14-Apr-12	16
21	1	1	1	1		14
.	.	0	0	0		
18	0	1	1	1		7
26	1	1	1	1		4
17	0	0	0	0		
.	.	0	0	0		
.	.	0	0	0		
20	1	1	1	1		15
13	0	0	0	0		
.	.	0	0	0		
.	.	0	0	0		
.	.	1	1	1		7
.	.	1	0	0		
		1	1	1	24-Mar-14	4
		1	1	1	20-Apr-14	22
		1	1	1	1-Aug-15	9
		0	0	0		
		0	0	0		
		0	0	0		
		0	0	0		
		0	0	0		
		0	0	0		
		1	1	1	6-Jun-11	8
		1	1	1	25-Apr-12	12
		1	1	1	31-Aug-12	15
		0	0	0		

		0	0	0		
		0	0	0		
		0	0	0		
		0	0	0		
		0	0	0		
		0	0	0		
		1	1	1	16-Sep-12	4
		0	0	0		
		0	0	0		
		0	0	0		
		0	0	0		
		1	1	1	20-Aug-12	19
		0	0	0		
		1	0	0		
		0	0	0		
		0	0	0		
		0	0	0		
		0	0	0		
		0	0	0		
		1	1	1	17-Dec-13	13
		0	0	0		
		0	0	0		
0		0	0	0		

carpamsiner	tigecmas48h	dias tigec	rifamp48 hrs	dias rifamp	tx.combinad	triple esquer
0	0	0	0	0	0	0
0	0	0	0	0	0	0
0	0		1	15	1	0
0	0		0		0	0
1	0		0		1	0
1	0		0		1	0
0	0		0		0	0
0	0		0		0	0
0	0		0		0	0
0	0		0		0	0
0	0		0		0	0
0	0		0		0	0
0	0		0		0	0
0	0		0		0	0
0	0		0		0	0
0	0		0		0	0
0	0		0		0	0
0	0		0		0	0
1	0		1	7	1	1
1	0		1	18	1	1
0	0		0		0	0
0	0		0		0	0
0	0		0		0	0
0	0		0		0	0
0	0		0		0	0
0	0		0		0	0
1	0		0		0	0
1	0		1	11	1	1
0	0		0		0	0
1	1	7	0		1	0
1	0		1	24	1	1
0	0		0		0	0
1	0		0		1	0
0	0		0		0	0
0	0		0		0	0
0	0		0		0	0
0	0		0		0	0
0	0		0		0	0
0	0		0		0	0
0	0		0		0	0
1	0		1	8	1	0

0	0		1	16	1	0
0	0		0		0	0
0	0		0		0	0
1	0		0		1	0
0	0		0		0	0
0	0		0		0	0
0	0		0		0	0
0	0		0		0	0
0	0		0		0	0
0	0		0		0	0
0	0		0		0	0
1	0		0		1	0
1	0		0		1	0
1	0		1	4	1	1
0	1	14	0		0	0
1	0		0		1	0
1	0		0		1	0
0	0		0		0	0
0	0		1	7	1	0
0	0		0		1	0
0	0		0		0	0
0	0		0		0	0
0	0		0		0	0
0	0		1	15	1	0
0	0		0		0	0
0	0		0		0	0
0	0		0		0	0
1	0		0		1	0
1	0		0		1	0
0	0		0		0	0
1	0		1	22	1	1
1	0		0		1	0
0	0		0		0	0
0	0		0		0	0
0	0		0		0	0
0	0		0		0	0
0	0		0		0	0
0	0		0		0	0
1	0		0		1	1
0	0		1	12	1	0
1	0		1	15	1	1
0	0		0		0	0

0	0	0		0	0
0	0	0		0	0
0	0	0		0	0
0	0	0		0	0
0	0	0		0	0
0	0	0		0	0
0	0	0		0	0
0	0	0		0	0
0	0	0		0	0
0	0	0		0	0
0	0	0		0	0
0	0	1	19	1	0
0	0	0		0	0
1	0	0		1	1
0	0	0		0	0
0	0	0		0	0
0	0	0		0	0
0	0	0		0	0
0	0	0		0	0
0	0	0		0	0
0	0	0		0	0
0	0	0		0	0
0	0	0		0	0

Abs acinetob	Dias Abs acir	Abs acineto2	Dias abs acir	abs acineto3	dias abs cine	VMA?
.	.	.	.	.	.	0
25	2	26	2	.	.	1
25	15	26	15	.	.	1
.	.	.	.	.	.	1
25	7	26	1	.	.	1
25	20	.	.	.	.	1
.	.	.	.	.	.	1
.	.	.	.	.	.	1
.	.	.	.	.	.	1
.	.	.	.	.	.	0
.	.	.	.	.	.	1
.	.	.	.	.	.	1
25	2	.	.	.	.	1
.	.	.	.	.	.	1
.	.	.	.	.	.	1
.	.	.	.	.	.	1
.	.	.	.	.	.	1
25	1	26	1	.	.	1
25	114	26	7	28	9	1
25	18	26	18	8	1	1
.	.	27	7	.	.	0
.	.	.	.	.	.	1
25	8	.	.	.	.	1
.	.	.	.	.	.	1
.	.	.	.	.	.	1
25	2	.	.	.	.	1
25	4	.	.	.	.	1
25	10	26	11	.	.	1
.	.	.	.	.	.	1
27	7	.	.	.	.	1
25	8	26	7	.	.	1
.	.	.	.	.	.	0
25	3	.	.	.	.	1
.	.	.	.	.	.	1
.	.	.	.	.	.	0
.	.	.	.	.	.	1
.	.	.	.	.	.	1
.	.	.	.	.	.	0
.	.	.	.	.	.	1
25	2	.	.	.	.	1
25	18	26	8	.	.	1

25	16	26	16	.	.	1
.	.	.	.	.	.	1
.	.	.	.	.	.	0
25	3	.	.	.	.	1
.	.	.	.	.	.	1
.	.	.	.	.	.	0
.	.	.	.	.	.	1
.	.	.	.	.	.	1
25	2	26	2	.	.	0
.	.	.	.	.	.	1
.	.	.	.	.	.	1
25	10	.	.	.	.	0
25	5	.	.	.	.	1
25	29	26	4	.	.	1
27	14	.	.	.	.	1
25	16	.	.	.	.	1
25	14	.	.	.	.	1
.	.	.	.	.	.	1
25	6	26	6	.	.	1
25	6	26	2	.	.	1
.	.	.	.	.	.	1
.	.	.	.	.	.	1
.	.	.	.	.	.	1
25	15	26	15	.	.	1
.	.	.	.	.	.	1
.	.	.	.	.	.	1
.	.	.	.	.	.	1
25	7	12	7	.	.	0
11	7	4	6	.	.	0
25	4	.	.	.	.	0
25	22	26	22	12	22	0
25	9	12	9	.	.	0
.	.	.	.	.	.	0
.	.	.	.	.	.	0
.	.	.	.	.	.	0
.	.	.	.	.	.	0
.	.	.	.	.	.	0
.	.	.	.	.	.	0
25	8	12	11	4	11	0
25	12	26	12	.	.	0
25	35	26	21	12	14	1
.	.	.	.	.	.	0

.	.	.	.	.	.	0
.	.	.	.	.	.	1
.	.	.	.	.	.	0
.	.	.	.	.	.	0
.	.	.	.	.	.	0
.	.	.	.	.	.	0
25	4	.	.	.	.	0
.	.	.	.	.	.	0
.	.	.	.	.	.	1
.	.	.	.	.	.	1
.	.	.	.	.	.	0
25	19	26	19	.	.	0
.	.	.	.	.	.	0
12	.	26	14	4	14	0
.	.	.	.			0
.	.	.	.	.	.	1
.	.	.	.	.	.	1
.	.	.	.	.	.	0
.	.	.	.	.	.	0
25	13	.	.	.	.	1
.	.	.	.	.	.	1
.	.	.	.	.	.	0
.	.	.	.	.	.	0

Fecha Inicio	Fecha termir	Dias VMA	diasiniciovm	diasfinvmaa	Cirugia?	Tpo. Qx.
.	.	.	.	.	0	.
28-Apr-13	3-May-13	5	0	5	0	.
11-Nov-12	30-Nov-12	7	3	16	0	.
29-Jul-12	1-Aug-12	2	1	2	1	1
26-Jan-12	11-Feb-12	16	7	9	0	.
1-Nov-11	4-Dec-11	34	6	27	1	.
20-Oct-11	30-Oct-11	10	13	-3	0	.
4-Nov-11	8-Nov-11	4	2	2	1	.
30-Sep-11	3-Oct-11	3	3	0	1	.
.	.	.	.	.	1	toractotomia
7-Feb-14	10-Feb-14	3	10	-7	0	.
2-Dec-11	11-Dec-11	9	6	3	0	.
19-May-11	29-May-11	10	7	3	0	.
27-Oct-12	5-Nov-12	9	0	9	1	whipple
2-Sep-12	11-Sep-12	9	8	1	0	.
2-Apr-11	9-Apr-11	7	2	5	0	.
12-Apr-14	13-Apr-14	1	-2	3	0	.
31-Mar-12	12-Apr-12	12	11	1	1	.
18-Feb-14	17-Mar-14	27	15	12	0	.
13-Oct-12	22-Nov-12	35	4	36	1	.
.	.	.	.	.	0	.
4-Oct-11	11-Oct-11	7	6	1	1	.
20-May-11	18-Jun-11	29	20	9	0	.
6-Aug-11	22-Aug-11	16	18	-2	0	.
25-Apr-11	8-May-11	13	13	0	0	.
3-Feb-14	3-Feb-14	1	-2	2	0	.
4-Feb-14	15-Feb-14	11	6	5	0	.
30-Jan-12	27-Feb-12	28	0	28	0	.
26-Jan-11	27-Feb-11	31	36	-4	0	.
20-Jan-12	20-Jan-12	1	14	-14	1	.
3-Jul-12	10-Aug-12	38	26	12	0	.
.	.	.	.	.	0	.
21-Jan-12	30-Jan-12	9	6	3	0	.
13-Jul-12	14-Jul-12	1	0	1	1	.
.	.	.	.	.	0	.
16-Aug-11	22-Aug-11	6	2	4	0	.
10-Jul-11	28-Jul-11	18	16	2	0	.
.	.	.	.	.	0	.
6-Mar-11	12-Mar-11	6	12	-6	0	.
8-Nov-11	28-Nov-11	25	9	11	0	.
11-Apr-12	8-May-12	25	9	18	0	.

11-Sep-12	13-Oct-12	35	9	23	1	nefrec der,la
21-Nov-11	22-Nov-11	1	-2	3	0	.
.	.	.	.	.	1	explorac cavi
2-Nov-15	5-Nov-15	3	-2	5	0	.
2-Sep-12	27-Sep-12	25	29	-4	1	lape
.	.	.	.	.	0	.
13-Jan-13	4-Feb-13	22	10	12	0	.
27-Dec-12	11-Jan-13	15	11	4	1	lape
.	.	.	.	.	0	.
27-May-11	9-Jun-11	13	11	2	0	.
26-Aug-11	30-Aug-11	4	4	0	1	craneotomia
.	.	.	.	.	0	.
6-Jan-12	4-Feb-12	29	19	10	0	.
29-Mar-12	24-Apr-12	26	12	14	1	esofagectom
14-Feb-12	13-Mar-12	27	20	8	0	.
2-Apr-12	16-May-12	44	9	35	0	.
24-Jun-14	7-Jul-14	13	25	-12	1	lape
26-Oct-11	5-Dec-11	41	21	-18	0	.
3-Jun-14	13-Jun-14	9	4	-6	0	.
15-May-11	2-Jun-11	18	10	-8	0	.
10-Jul-14	11-Jul-14	1	-1	-2	0	.
29-Jul-12	30-Jul-12	3	-1	2	0	.
10-Dec-12	3-Jan-13	23	32	9	0	.
23/08/2013y	6-sep-13 a-2(	12	22	18	0	.
23-Feb-11	31-Mar-11	36	43	7	0	.
20-Apr-11	30-Apr-11	10	8	-2	0	.
29-Apr-11	30-Apr-11	1	-1	-2	0	.
.	.	.	.	.	0	.
.	.	.	.	.	0	.
.	.	.	.	.	0	
.	.	.	.	.	0	.
.	.	.	.	.	0	.
.	.	.	.	.	0	.
.	.	.	.	.	0	.
.	.	.	.	.	1	resec. Tibia
.	.	.	.	.	1	exenterac pe
.	.	.	.	.	1	exenterac pe
.	.	.	.	.	0	.
.	.	.			0	.
.	.	.	.	.	0	.
22-Aug-12	2-Oct-12	41	7	-33	0	.
.	.	.	.	.	1	lape

.	.	.	.	.		0	.
22-Oct-11	5-Nov-11	14	14	1		1	tiroidectomia
.	.	.	.	.		0	.
.	.	.	.	.		0	.
.	.	.	.	.		0	.
.	.	.	.	.		1	resec condro
.	.	.	.	.		0	
.	.	.	.	.		0	.
6-Jun-11	19-Jun-11	13	22	9		1	lape + empac
14-Aug-12	20-Aug-12	6	-3	-9		1	lape + resec i
.	.	.	.	.		0	.
.	.	.	.	.		1	hemipelvecto
.	.	.	.	.		1	lape + hemip
						0	
.	.					0	.
19-Feb-11	1-Apr-11	41	36	-5		0	.
22-Dec-12	23-Dec-12	1	1	0		0	.
.	.	.	.	.		0	.
.	.	.	.	.		0	.
27-Dec-13	30-Dec-13	3	-22	-25		0	.
13-Feb-13	22-Feb-13	9	21	9		0	.
.	.	.	.	.		0	.
.	.	.	.	.		0	.

Fecha egreso	diasacinetoe	Motivo egres	Evol 30 dias	Evol. 12 mes	Fecha ultima	diasacinetou
15-Jun-11	1	2	2	2	15-Jun-11	1
3-May-13	5	2	2	2	3-May-13	5
7-Dec-12	23	1	1	1	6-Nov-13	357
1-Aug-12	2	2	2	2	1-Aug-12	2
11-Feb-12	9	2	2	2	11-Feb-12	9
21-Dec-11	44	1	1	1	9-Aug-13	641
15-Nov-11	13	1	1	1	29-May-15	1304
11-Nov-11	5	1	1	1	31-May-16	1668
7-Oct-11	4	1	1	.	19-Jun-12	260
21-Sep-11	7	1	1	1	20-Oct-16	1863
17-Feb-14	0	2	2	2	17-Feb-14	0
11-Dec-11	3	2	2	2	11-Dec-11	3
29-May-11	3	4	4	4	29-May-11	3
4-Nov-12	8	1	2	2	5-Nov-12	9
11-Sep-12	1	2	2	2	11-Sep-12	1
9-Apr-11	5	3	3	3	9-Apr-11	5
13-Apr-14	3	2	2	2	13-Apr-14	3
12-Apr-12	1	2	2	2	12-Apr-12	1
25-Mar-14	20	4	4	4	25-Mar-14	20
22-Nov-12	36	4	4	4	22-Nov-12	36
6-Aug-11	24	1	1	2	13-Oct-11	92
14-Oct-11	4	1	1	.	9-Nov-14	1126
18-Jun-11	9	2	2	2	18-Jun-11	9
30-Aug-11	6	1	1	1	11-Sep-17	2210
8-May-11	0	2	2	2	8-May-11	0
3-Feb-14	2	2	2	2	3-Feb-14	2
15-Feb-14	5	2	2	2	15-Feb-14	5
16-Apr-12	77	1	1	desc	3-Jul-12	155
18-Mar-11	15	5	5	5	18-Mar-11	15
17-Feb-12	14	2	2	2	17-Feb-12	14
10-Aug-12	12	2	2	2	10-Aug-12	12
26-Jun-12	3	2	2	2	26-Jun-12	3
30-Jan-12	3	2	2	2	30-Jan-12	3
14-Jul-12	1	2	2	2	14-Jul-12	1
6-Jun-13	3	3	3	3	6-Jun-13	3
22-Sep-11	35	2	2	2	22-Aug-11	4
22-Aug-11	27	4	4	4	22-Aug-11	27
10-Mar-13	1	2	2	2	10-Mar-13	1
24-Mar-11	6	1	1	1	10-Apr-12	389
9-Dec-11	22	2	2	2	9-Dec-12	388
8-May-12	18	2	2	2	8-May-12	18

13-Oct-12	23	2	2	2	13-Oct-12	23
22-Nov-11	3	2	2	2	22-Nov-11	3
1-Sep-13	0	2	2	2	1-Sep-13	0
5-Nov-15	5	2	2	2	5-Nov-15	5
12-Nov-12	42	1	1	1	2-Jan-14	458
6-Feb-14	9	1	1	nd	10-Nov-14	286
3-Apr-13	70	1	1	nd	25-May-13	122
14-Feb-13	38	3	3	3	14-Feb-13	38
20-Aug-13	4	2	2	2	20-Aug-13	4
16-Jun-11	9	4	4	4	16-Jun-11	9
1-Sep-11	2	1	1	3	22-Nov-11	84
9-May-11	18	1	1	3	30-Jun-11	70
4-Feb-12	10	2	2	2	4-Feb-12	10
16-May-12	36	1	1	5	6-Jul-12	87
6-Apr-12	32	1	1	5	12-Dec-12	282
1-Jun-12	51	1	5	3	5-Feb-13	300
12-Aug-14	24	1	3	3	29-Aug-14	41
6-Dec-11	19	3	3	3	6-Dec-11	19
13-jun-14	6	2	2	2	13-Jun-14	6
02-jun-11	8	3	3	3	2-Jun-11	8
11-Jul-14	2	2	2	2	11-Jul-14	2
30-Jul-12	2	2	2	2	30-Jul-12	2
20-Jan-13	9	1	1	1	3-Sep-18	2061
20-Sep-13	5	2	2	2	20-Sep-13	5
16-Apr-11	9	1	1	1	23-Aug-12	494
30-Apr-11	2	2	2	2	30-Apr-11	2
30-Apr-11	2	2	2	2	30-Apr-11	2
20-Jun-16	9	1	1	4	23-Jan-17	221
27-Jun-11	11	1	1	3	27-Jun-11	10
28-Mar-14	13	1	1	3	22-Sep-14	191
12-Jun-14	26	1	3	3	12-Jun-14	56
11-Aug-15	15	1	1	1	9-Aug-16	379
.	.	1	3	3	17-Oct-11	6
20-Apr-12	0	1	1	3	5-Jun-12	47
21-May-12	3	1	1	1	22-Jan-16	1344
5-Jun-12	11	1	1	1	9-Nov-16	1629
10-May-12	1	1	1	1	2-Oct-19	2702
18-May-12	22	1	3	3	18-May-11	23
18-Jun-11	13	1	1	1	23-Sep-19	3032
8-May-12	33	1	1	3	19-Oct-12	197
31-Oct-12	63	1	1	3	7-Jul-13	312
21-Aug-11	-10	1	1	3	29-Dec-11	120

7-Sep-11	14	1	1	3	24-Apr-12	244
13-Nov-11	7	1	1	1	8-Dec-12	398
3-Feb-12	1	1	1	3	6-Jun-12	125
15-Sep-11	4	3	3	3	15-Sep-11	4
.	.	1	1	1	15-Oct-19	3007
23-Aug-12	0	1	1	1	29-Jun-15	1040
29-Sep-12	15	3	3	3	29-Sep-12	15
27-Sep-12	2	1	1	1	20-Feb-13	148
7-Jul-11	9	1	1	3	15-Jul-11	17
8-Sep-12	28	1	1	3	2-Oct-12	52
12-Jun-11	6	3	3	3	12-Jun-11	6
22-Sep-12	40	1	1	3	22-Sep-12	39
4-Nov-12	39	1	1	1	2-Sep-14	667
5-Jun-11	22	1	1	1	23-May-13	741
12-Apr-11	10	4	3	3	14-Apr-11	12
13-Apr-11	17	1	1	1	3-Jun-19	2990
23-Dec-12	0	2	2	2	23-Dec-12	0
12-Jan-13	8	4	4	4	12-Jan-13	8
31-Mar-14	2	4	3	3	3-Apr-14	5
30-Dec-13	25	3	3	3	30-Dec-13	25
3-Mar-13	0	1	1	1	14-Nov-18	2082
.	.	1	1	1	26-Apr-13	193
11-Dec-12	8	1	3	3	16-Dec-12	13

semacinetult	vivomto30di	vivomto72hr	choque xacir	tw	Perfil	causainguci
0.14	1	1		2011w9	V	.
0.71	1	0	1	2011w14	V	3
51.00	0	0		2011w14	V	4
0.29	1	1		2011w16	V	7
1.29	1	0	0	2011w17	V	3
91.57	0	0		2011w19	V	2
186.29	0	0	0	2011w19	V	3
238.29	0	0		2011w21	V	1
37.14	0	0		2011w24	V	2
266.14	0	0		2011w24	V	4
0.00	1	1	0	2011w28	V	3
0.43	1	1		2011w33	V	2
0.43	1	1		2011w34	V	7
1.29	1	0	1	2011w37	V	3
0.14	1	1		2011w40	V	4
0.71	1	0	1	2011w41	V	3
0.43	1	1	0	2011w45	V	3
0.14	1	1		2011w47	V	4
2.86	1	0		2011w49	V	4
5.14	1	0		2012w4	XXI	1
13.14	0	0		2012w5	V	.
160.86	0	0		2012w10	V	2
1.29	1	0	0	2012w15	V	3
315.71	0	0		2012w15	V	2
0.00	1	1	0	2012w15	V	3
0.29	1	1	1	2012w16	V	3
0.71	1	0		2012w17	V	4
22.14	0	0	1	2012w18	V	3
2.14	0	0		2012w20	V	4
2.00	1	0		2012w21	V	1
1.71	1	0		2012w25	V	4
0.43	1	1		2012w28	V	.
0.43	1	1		2012w31	V	4
0.14	1	1		2012w31	V	4
0.43	1	1		2012w32	V	.
0.57	1	0		2012w33	V	4
3.86	1	0		2012w34	V	4
0.14	1	1		2012w37	V	.
55.57	0	0	0	2012w38	V	3
55.43	1	0	0	2012w39	V	3
2.57	1	0		2012w40	V	4

3.29	1	0		2012w42	V	2
0.43	1	1	1	2012w43	V	3
0.00	1	1	1	2012w46	V	3
0.71	1	0		2012w49	V	7
65.43	0	0	0	2013w4	V	3
40.86	0	0		2013w9	V	.
17.43	0	0		2013w10	V	4
5.43	1	0	0	2013w33	V	3
0.57	1	0		2013w35	V	.
1.29	1	0		2014w4	V	4
12.00	0	1		2014w5	V	1
10.00	0	0		2014w7	V	.
1.43	1	0		2014w11	V	4
12.43	0	0	0	2014w13	V	3
40.29	0	0	0	2014w15	V	3
42.86	0	0		2014w16	V	2
5.86	1	0	0	2015w30	V	3
2.71	1	0			XIX	7
0.86	1	0	0		XVI	3
1.14285714	1	0			XVIII	1
0.3	1	1			XVI	.
0.3	1	1			X	.
294.4	0	0			XII	1
0.7	1	0			XVI	4
70.6	1	1			V	4
0.3	1	1	0		V	3
0.3	1	1			V	.
31.6	1	0	0	2016	XVII	.
1.3	0	0	0	2011	VIII	.
37.3	0	0	0		V	.
8	1	0	0	2014	V	.
54.1	0	0	0	2015w30	V	.
0.8	1	1	0	.	VIII	.
6.7	0	0	0		V	.
192	0	0	0		V	.
232.7	0	0	0		V	.
386	0	0	0		V	.
3.2	0	0	0		V	.
433.1	0	0	0		V	.
28.1	0	0	0		V	1
44.4	0	0	0		V	3
17.1	0	0	0		V	.

34.8	0	0	0		V	.
56.8	0	0	0		V	7
17.8	0	0	0		V	.
0.6	1	0	0		V	.
429.6	0	0	0		V	.
148.6	0	0	0		V	.
2.1	1	0	0		V	.
21.1	0	0	0		V	.
2.4	0	0	0		V	1
7.3	0	0	0		V	1
0.9	1	0	0		V	.
5.7	0	0	0		V	.
95.3	0	0	0		V	.
105.8	0	0	0		V	
1.5	1	0	0		V	.
427.1	0	0	0		V	4
0	0	0	1		V	3
1.1	1	0			V	.
0.7	0	0	0		V	
3.6	0	0	0		V	4
297.4	0	0	0		V	3
27.6	0	0	0		V	.
2	0	0	0		V	.

diashospitacinet
7
31
6
5
10
7
26
3
4
7
18
8
14
1
71
4
3
14
16
6
28
7
37
23
13
9
19
15
41
17
30
19
12
11
3
22
21
17
14
13
23

11
23
18
9
35
-15
36
18
7
12
4
29
19
19
18
15
38
42
22
10
27
13
35
26
54
12
15
-366
12
4
0
0
.
12
0
2
9
57
3
11
44
14

2
17
6
4
.
8
1
2
23
-1
0
-7
23
30
25
39
0
9
4
-12
19
.
4
