# Supplementary material for: High mortality in an outbreak of multidrug resistant Acinetobacter baumannii infection introduced to an oncological hospital by a patient transferred from a general hospital
Source: PLoS One. 2020 Jul 23;15(7):e0234684. doi: 10.1371/journal.pone.0234684 (PMC7377454; doi:10.1371/journal.pone.0234684)
Supplement: Attachment — Submitted filename: Answer letter Reviewers Plow One March 2020.docx [file pone.0234684.s003.docx]

PONE-D-19-33916.

High mortality in an outbreak of multidrug resistant Acinetobacter baumannii introduced to an oncological hospital by a patient transferred from a general hospital.

In your revised cover letter, please address the following prompts:

a) If there are ethical or legal restrictions on sharing a de-identified data set, please explain them in detail (e.g., data contain potentially identifying or sensitive patient information) and who has imposed them (e.g., an ethics committee). Please also provide contact information for a data access committee, ethics committee, or other institutional body to which data requests may be sent.

There is not an ethical restriction on sharing a de-identified data set. Contact information is: Ethics and Investigation Committee. Instituto Nacional de Cancerologia. Dr. Myrna Candelaria Hernández. San Fernando No. 22, Col. Sección XBI, Tlalpan, Mexico City. Mexico. Tel: +52-55-56280400 ext.37015.

b) If there are no restrictions, please upload the minimal anonymized data set necessary to replicate your study findings as either Supporting Information files or to a stable, public repository and provide us with the relevant URLs, DOIs, or accession numbers. Please see http://www.bmj.com/content/340/bmj.c181.long for guidelines on how to de-identify and prepare clinical data for publication. For a list of acceptable repositories, please see <http://journals.plos.org/plosone/s/data-availability#loc-recommended-repositories>.

We uploaded anonymized data set.

Reviewer 1. The manuscript entitled “High mortality in an outbreak of multidrug resistant *Acinetobacter baumannii* infection introduced to an oncological hospital by a patient transferred from a general hospital.” By Volkow-Fernández et al. reports an outbreak of multidrug resistant (MDR A. baumannii).

In my opinion the manuscript is of interest from an epidemiological and clinical point of view but the introduction, methods and discussion section are limited, I recommend presenting this manuscript in a better fashion. There are many errors in the documented presented.

1. “We analyzed all MDR A. baumannii strains”. How many?

We included in methods the following (page 4, 2nd paragraph)

*In March 2011, we isolated the first strain of MDR A. baumannii; since then, multiple isolates from different sources of hospitalized patients at the ICU were recovered. No MDR A. baumanii strains had been isolated previously in the hospital. During 2011 to 2012, 78 isolates were identified (73.6%), decreasing the number of isolates recovered in the coming three years, until 2015.*

2. During the study period, 108 patients with MDR A. baumannii isolates were identified 1 strain per patient?

We completed the phrase to make clear that was one strain per patient included (Page 4 line 11-12):

*From January 2011 to December 2015, 106 patients with MDR A. baumannii isolates were identified, all were included in the study.*

3. Fig. 1a and 1b. No figures in the manuscript.

We checked and all figures were included in the built pdf form at submission.

4. “Of all the isolates obtained during the outbreak” How many?

This information was incorporated in the methods section (page 4, line 11) also commented in in the answer of question 2

5. The antibiotic resistance profile of the strains?

This information was incorporated in the text (Page 6, first paragraph).

*All the strains included in this study, were resistant to all previous antibiotics families except tetracyclines and colistin.*

6. Did you know what kind of mechanism of resistance was behind the strains?

*No, we have not investigated it.*

7. Please provide a comment in the discussion about detection of these strains in the micro lab and infection control. What interventions were made in response to the outbreak.

A comment was included about the detection in the microbiology laboratory and the measures taken to control the outbreak.

We incorporated in page 18, 2^nd^ paragraph.:

*During the outbreak, prevention measures were implemented and others were reinforced; the measures included: a single nurse per patient, a single cleaning material quit per each room, exhaustive daily cleaning of all ventilation equipment, hand hygiene program reinforcement, strict contact isolation, clorhexidine oral washing three times per day, and patient daily bathing with clorhexidine. Antimicrobial control policies were implemented in year 2013. Also, for patients transferred from other hospital initial cultures from nares, axillary, perineal region and draining sites were taken. All the measures contributed to end the outbreak in year 2014. Environmental and tap water surveillance was also performed during the outbreak period (44 samples taken in 2011, and 111 in 2012). Only one tap water sample (isolated in Feb 2012) was positive for MDR A. baumanniii ST758 profile V at the hematopoietic stem cell transplant unit. No patient in this unit was diagnosed with MDR A. baumanniii infection.*

8. In colistin and tigecycline treatment please include the dose and time according site of infection.

The information was included (Page 13, 2^nd^ paragraph).

*The mean days for colistin, the mean days were 12 ± 6 days (no differences between type of infection). Two patients received tigecycline (one as monotherapy and one combined with meropenem), for 7 and 14 days each one.*

6. Please include in the results and discussion the different MLST found in the study.

We have incorporated MLST findings in the results section (page 8, 2nd paragraph):

*Of all the isolates obtained during the outbreak, 99 had the same sequence type MLST 758; 94 had the same plasmid profile as the one recovered from the index case, two had the same plasmid an extra plasmid, and two others were also MLST 758 but had different plasmid profiles. Seven had different MLST (four of them proceeded from infected patients and had been hospitalized in another hospital within the previous 60 days).*

*In the discussion section (Page 16, 2^nd^ paragraph:*

*In this series, we included 99 patients with an MLST 758 strain: 95 with the same plasmid profile (V), two with plasmid XVI- very similar to V, and two with another plasmid profile. The remaining seven isolates belonged to different MLSTs with different plasmids patterns; all of them have been hospitalized recently. They were probably colonized with the MDR strain when they arrived, and it became invasive during hospitalization due to therapeutic procedures.*

*This MLST has been identified previously in Mexican hospitals and in other countries of Latin America. The MLST758 is an understudied lineage that does not belong to the international clones I and II; it has spread to Canada, Mexico, Honduras, and Colombia. This lineage represents a source of genetic diversity of MDR and XDR isolates that have not been studied [12, 13].*

7. Please rewrite limitations of the study

We incorporate a new version of the limitations of the study. (Page 19, last paragraph).

*The study has some limitations: we included patients from a single tertiary-care referral cancer center, that does not allow to extrapolate the data to other scenarios. We also were not able to get information from resistance isolates of the hospital where the index patient was hospitalized previous to the transfer to our institution; we were able to establish through molecular epidemiology the original source of the strain.*

*However, the strengths are that it allows establishing some risk factors associated with infection and mortality from this pathogen, and we were able to demonstrate the clonal relation in most all the strains of this outbreak.*

Reviewer #2: In this manuscript, the authors describe an outbreak of multidrug resistant Acinetobacter baumannii infection in a tertiary care cancer hospital in Mexico City. They describe the clinical features, outcomes, and molecular epidemiology of this outbreak.

Major comments
The clinical part of this work is complete. However, the absence of data concerning the antibiotic resistance profile for each strain is regrettable and must be shown since the antimicrobial susceptibility was determined, as described in the Methods part.

1. The information with the susceptibility was reviewed and some information was incorporated (Page 5, last paragraph).

*All the strains included in this study, were resistant to all previous antibiotics families except tetracyclines and colistin.*

2. Furthermore, also regrettable, there is no figure with the experimental results of plasmid profile (agarose gels). It will be of interest to visualize these different profiles.

*The picture of an agarose gel showing the different profiles was included as figure 2..*

Specific comments
3. It is stated in the abstract that “we documented an outbreak of an MDR A. baumannii strain identified initially in 2011 ... “ : Is there any clear evidence that all the strains isolated until 2015 are related to the index case ?

*The same strain with the same plasmid profile isolated from the index case, that was a patients that had been transferred from a General Hospital to our Institution, was found in 100% of the isolates from the years 2011 to 2012, and in 93.4% from the whole strains.*

4. It could be of interest to show the relation between clinical data (localization, classification (infected or colonized), malignancies) and the sequence typing of the strain.

*Ninety-nine of 106 strains had the same MLST, so only 7 patients had a different strain, we considered that the group is too small to do an analysis.*

5. Why do the authors performed univariate and multivariate analysis ?

*The uni- and multivariate analyses were performed to assess clinical risk factors related to mortality and for colonization vs. infection.*

6. At the end of the Results part, it is stated that “98 had the same sequence type ST758”. However, in the S1 table, it appears that 99 strains exhibit this ST. Could the authors clarify this point ?

There were 98 strains plus the index case, as the way it was written created confusion, we have modified the manuscript.

In the abstract section:

*The index case, identified by molecular epidemiology, was a patient with a drain transferred from a hospital outside Mexico City. Ninety-eight additional cases had the same (MLST 758, of which 94 also had the same plasmid profile, two had an extra plasmid, and two had a different plasmid. The remaining seven isolates belonged to different MLSTs.*

*In the result section (Page 8, 2nd paragraph):*

*Of all the isolates obtained during the outbreak, 99 had the same sequence type MLST 758; 94 had the same plasmid profile as the one t recovered from the index case, two had the same plasmid an extra plasmid, and two others were also MLST 758 but had different plasmid profiles. Seven had different MLST (four of them proceeded from infected patients and had been hospitalized in another hospital within the previous 60 days).*

7. The quality of the figures is poor. Furthermore, a detailed legend must be added for each figure.

A new version of the figure much clearer with the corresponding figure legend (page 8, 3^rd^ paragraph).
