# Supplementary material for: High mortality in an outbreak of multidrug resistant Acinetobacter baumannii infection introduced to an oncological hospital by a patient transferred from a general hospital
Source: PLoS One. 2020 Jul 23;15(7):e0234684. doi: 10.1371/journal.pone.0234684 (PMC7377454; doi:10.1371/journal.pone.0234684)
Supplement: Attachment — Submitted filename: PLOs One- letter 2nd review April 2020.docx [file pone.0234684.s004.docx]

**View Letter**

| **Date:** | Apr 02 2020 08:05AM |
| --- | --- |
| **To:** | "Patricia Volkow-Fernández" pcornejoj@gmail.com |
| **From:** | "PLOS ONE" plosone@plos.org |
| **Subject:** | PLOS ONE Decision: Revision required [PONE-D-19-33916R1] |

PONE-D-19-33916R1
High mortality in an outbreak of multidrug resistant Acinetobacter baumannii introduced to an oncological hospital by a patient transferred from a general hospital.
PLOS ONE

Dear Dra. Volkow-Fernández,

Thank you for submitting your manuscript to PLOS ONE. After careful consideration, we feel that it has merit but does not fully meet PLOS ONE’s publication criteria as it currently stands. Therefore, we invite you to submit a revised version of the manuscript that addresses the points raised during the review process.

Your revisions have significantly improved this manuscript, however, they are asking for more. We would appreciate receiving your revised manuscript by May 17 2020 11:59PM. When you are ready to submit your revision, log on to https://www.editorialmanager.com/pone/ and select the 'Submissions Needing Revision' folder to locate your manuscript file.

If you would like to make changes to your financial disclosure, please include your updated statement in your cover letter.

To enhance the reproducibility of your results, we recommend that if applicable you deposit your laboratory protocols in protocols.io, where a protocol can be assigned its own identifier (DOI) such that it can be cited independently in the future. For instructions see: <http://journals.plos.org/plosone/s/submission-guidelines#loc-laboratory-protocols>

Please include the following items when submitting your revised manuscript:

- A rebuttal letter that responds to each point raised by the academic editor and reviewer(s). This letter should be uploaded as separate file and labeled 'Response to Reviewers'.
- A marked-up copy of your manuscript that highlights changes made to the original version. This file should be uploaded as separate file and labeled 'Revised Manuscript with Track Changes'.
- An unmarked version of your revised paper without tracked changes. This file should be uploaded as separate file and labeled 'Manuscript'.

Please note while forming your response, if your article is accepted, you may have the opportunity to make the peer review history publicly available. The record will include editor decision letters (with reviews) and your responses to reviewer comments. If eligible, we will contact you to opt in or out.

We look forward to receiving your revised manuscript.

Kind regards,

Monica Cartelle Gestal, PhD
Academic Editor
PLOS ONE


[Note: HTML markup is below. Please do not edit.]

Reviewers' comments:

Reviewer's Responses to Questions

**Comments to the Author**

1. If the authors have adequately addressed your comments raised in a previous round of review and you feel that this manuscript is now acceptable for publication, you may indicate that here to bypass the “Comments to the Author” section, enter your conflict of interest statement in the “Confidential to Editor” section, and submit your "Accept" recommendation.

Reviewer #1: (No Response)

Reviewer #2: All comments have been addressed

2. Is the manuscript technically sound, and do the data support the conclusions?

The manuscript must describe a technically sound piece of scientific research with data that supports the conclusions. Experiments must have been conducted rigorously, with appropriate controls, replication, and sample sizes. The conclusions must be drawn appropriately based on the data presented.

Reviewer #1: Partly

Reviewer #2: Yes

3. Has the statistical analysis been performed appropriately and rigorously?

Reviewer #1: Yes

Reviewer #2: N/A

4. Have the authors made all data underlying the findings in their manuscript fully available?

The [PLOS Data policy](http://www.plosone.org/static/policies.action#sharing) requires authors to make all data underlying the findings described in their manuscript fully available without restriction, with rare exception (please refer to the Data Availability Statement in the manuscript PDF file). The data should be provided as part of the manuscript or its supporting information, or deposited to a public repository. For example, in addition to summary statistics, the data points behind means, medians and variance measures should be available. If there are restrictions on publicly sharing data—e.g. participant privacy or use of data from a third party—those must be specified.

Reviewer #1: No

Reviewer #2: Yes

5. Is the manuscript presented in an intelligible fashion and written in standard English?

PLOS ONE does not copyedit accepted manuscripts, so the language in submitted articles must be clear, correct, and unambiguous. Any typographical or grammatical errors should be corrected at revision, so please note any specific errors here.

Reviewer #1: No

Reviewer #2: Yes

6. Review Comments to the Author

Please use the space provided to explain your answers to the questions above. You may also include additional comments for the author, including concerns about dual publication, research ethics, or publication ethics. (Please upload your review as an attachment if it exceeds 20,000 characters)

Reviewer #1: In my opinion, the authors solved many of the questions and the manuscript is of interest from a clinical and epidemiological point of view. However, it is important to improve the manuscript in scientific content and the presentation to cover the standards required for publication.

1. Please, I recommend to include line numbers

We included line numbers.

2. [5]. “This pathogen is able to grow at various temperatures and pH conditions, so it has the ability to persist in either moist or dry conditions in the hospital environment, thereby contributing to transmission” [5] Please include more references in the study and avoid same citation in a paragraph.

*Answer: We have included 3 references related to the capacity of the pathogen to survive and multiple in diverse conditions. Page 3. Line 67.*

- *Baumann P. Isolation of Acinetobacter from soil and water. J Bacteriol. 1968;96(1):39–42*
- *Warskow AL, Juni E. Nutritional requirements of Acinetobacter strains isolated from soil, water, and sewage. J Bacteriol. 1972;112(2):1014–1016.*
- *Jawad A, Heritage J, Snelling AM, Gascoyne-Binzi DM, Hawkey PM. Influence of relative humidity and suspending menstrua on survival of Acinetobacter spp. on dry surfaces. J Clin Microbiol. 1996;34(12):2881–2887.*

3. “identification and antimicrobial susceptibility were determined using the Gram-Negative Identification Panel (BDPhoenixTM automated system, New Jersey, USA).” Please include the MIC values

MICs values were included. Page 6, lines 126-128.

4. “DNA was extracted from a representative strain of each patter” How many AMR patterns did you find?

There was the same antimicrobial resistance pattern in all MLST758 samples (n=99, 93.4%). In the remaining 7 strains, there was a minimal difference in the MICs of different antibiotics, including quinolones, beta-lactams, and carbapenems, however they were considered resistant according to CLSI.

5. “A typical pulsed-field gel electrophoresis analysis of selected isolates of A. baumannii and representative plasmid profiles is shown in figure 2.” Please include PFGE in methods section.

There was a mistake in this statement, because we did not perform PFGE. The sentence was changed by: “Representative plasma profiles of selected Acinetobacter strains are shown in figure 2.” Page 8, line 179.

6. “First, rpoB’s Zone-1[9] was amplified to confirm taxonomic status” Amplified and sequenced?

Yes, amplified and sequenced. Sequenced was added in the sentence. Page 6, line 132. A method to obtain plasmid profiles from Acinetobacter was included as Supplement.

7. This MLST has been identified previously in Mexican hospitals and in other countries of Latin America. Please include more references to improve the discussion.
We included three references in this part. Page 16, line 270.

- Alcántar-Curiel MD, Rosales-Reyes R, Jarillo-Quijada MD, et al. Carbapenem-Resistant *Acinetobacter baumannii* in Three Tertiary Care Hospitals in Mexico: Virulence Profiles, Innate Immune Response and Clonal Dissemination. *Front Microbiol* 2019; 10: 2116. Published 2019 Sep 20. doi:10.3389/fmicb.2019.02116

- Tamayo-Legorreta EM, Garza-Ramos U, Barrios-Camacho H, et al. Identification of OXA-23 carbapenemases: novel variant OXA-239 in Acinetobacter baumannii ST758 clinical isolates in Mexico. *New Microbes New Infect* 2014; 2: 173–4. doi:10.1002/nmi2.60

- Vanegas JM, Higuita LF, Vargas CA, Cienfuegos AV, Rodríguez EA, Roncancio GE, Jiménez JN. [Carbapenem-resistant Acinetobacter baumannii causing osteomyelitis and infections of skin and soft tissues in hospitals of Medellín, Colombia]. Biomedica 2015; 35:522–30.

8. It would be important to characterize the resistance mechanisms found in ST758, and if possible, to sequence the strain and the plasmids that will show relevant information to complement the clinical part of the study.

Thanks for the comment. We have very recently published a manuscript (see reference below) where we not only characterised the resistance mechanisms in ST758 but also studied the phylogeography of this lineage. Of note, in this study more than 20 ST758 genomes were considered. To enrich our manuscript, we have included a couple of sentences in the discussion (page 18, first paragraph) talking about the resistance mechanisms found in ST758.

Recently, employing a population genomics approach, we described the emergence and dispersion of the lineage ST758, and also studied the antibiotic resistance mechanisms present in strains from this ST (REF mSphere article). This lineage emerged rather recently (about 22 years ago) and since then it has spread in many countries in Latin and North America; notably, strains from this lineage are not only carbapenem resistant but also multidrug resistant. In terms of acquired oxacillinases (OXAs), this lineage presents the worldwide-distributed OXA-23-like family but also the less disseminated OXA-40-like family. Page 18, lines 298-304.

The reference was included: *Graña-Miraglia L, Evans BA, López-Jácome LE, et al. Origin of OXA-23 Variant OXA-239 from a Recently Emerged Lineage of Acinetobacter baumannii International Clone V. mSphere. 2020;5(1):e00801-19. Published 2020 Jan 8. doi:10.1128/mSphere.00801-19*

Reviewer #2: (No Response)

7. PLOS authors have the option to publish the peer review history of their article. If published, this will include your full peer review and any attached files.


If you choose “no”, your identity will remain anonymous but your review may still be made public.


**Do you want your identity to be public for this peer review?** For information about this choice, including consent withdrawal, please see our [Privacy Policy](https://www.plos.org/privacy-policy).

Reviewer #1: No

Reviewer #2: No


[NOTE: If reviewer comments were submitted as an attachment file, they will be attached to this email and accessible via the submission site. Please log into your account, locate the manuscript record, and check for the action link "View Attachments". If this link does not appear, there are no attachment files to be viewed.]


While revising your submission, please upload your figure files to the Preflight Analysis and Conversion Engine (PACE) digital diagnostic tool, <https://pacev2.apexcovantage.com/>. PACE helps ensure that figures meet PLOS requirements. To use PACE, you must first register as a user. Registration is free. Then, login and navigate to the UPLOAD tab, where you will find detailed instructions on how to use the tool. If you encounter any issues or have any questions when using PACE, please email us at [figures@plos.org](mailto:figures@plos.org). Please note that Supporting Information files do not need this step.
